# Biobehavioral Mechanisms of Mindfulness as a Treatment for Chronic Stress: An RDoC Perspective

**DOI:** 10.1177/2470547017711912

**Published:** 2017-06-22

**Authors:** Eric L. Garland, Adam W. Hanley, Anne K. Baker, Matthew O. Howard

**Affiliations:** 1 Center on Mindfulness and Integrative Health Intervention Development (C-MIIND) College of Social Work, University of Utah, Salt Lake City, UT, USA; 2 School of Social Work, University of North Carolina at Chapel Hill, Chapel Hill, NC, USA

**Keywords:** amygdala, allostatic load, attention network, default mode network, habit systems, hypothalamic–pituitary–adrenal axis, mindfulness-to-meaning, reappraisal, stress, sustained threat

## Abstract

Mindfulness-based interventions have been heralded as promising means of alleviating chronic stress. While meta-analyses indicate that mindfulness-based interventions significantly reduce global measures of stress, how mindfulness-based interventions modulate the specific mechanisms underpinning chronic stress as operationalized by the National Institute of Mental Health research domain criteria (RDoC) of sustained threat has not yet been detailed in the literature. To address this knowledge gap, this article aims to (1) review evidence that mindfulness-based interventions ameliorate each of the 10 elements of behavioral dysregulation characterizing sustained threat via an array of mindful counter-regulatory strategies; (2) review evidence that mindfulness-based interventions modify biological domains implicated in sustained threat, such as the hypothalamic–pituitary–adrenal axis, as well as brain circuits involved in attentional function, limbic reactivity, habit behavior, and the default mode network; and (3) integrate these findings into a novel conceptual framework of mindful self-regulation in the face of stress—the Mindfulness-to-Meaning Theory. Taken together, the extant body of scientific evidence suggests that the practice of mindfulness enhances a range biobehavioral factors implicated in adaptive stress coping and induces self-referential plasticity, leading to the ability to find meaning in adversity. These mechanistic findings can inform the treatment development process to optimize the next generation of mindfulness-based interventions for greater therapeutic efficacy.

The adaptation of self to world entails stress, insofar as the environment presents perturbations to which the individual must respond by dynamically adjusting internal parameters to preserve biological and cognitive coherence. These adjustments are conserved and maximized through biobehavioral feedback loops that produce stability (negative feedback) or plasticity (positive feedback) in the pattern of response to the stressor. Through such cybernetic feedback processes, information about the relevance of the stressor to the self dynamically shapes the stress response and is crucial to self-regulation in the face of the stressor. This article will explore the role of mindfulness as a self-regulatory strategy in adaptation to chronic stress.

In classical^1^ and modern transactional models,^2–4^ successful allostatic adaptation to environmental perturbations (i.e., stressors) depends on a process of appraising goodness of fit between present situational demands, the current state of the individual, and prior learning. Hence, the individual appraises the stressor for its significance to the self-in-context and ascribes meaning to the stressor event relative to the individual’s ongoing autobiographical narrative. Such stress appraisals are often rapid and automatic,^5^ though they may be consciously mediated. If the individual deems the stressor to be a threat or harm that exceeds their resources, this determination initiates the acute stress reaction, which may result in stereotyped, habitual defensive behavior or volitional coping responses intended to preserve the integrity of the self or promote homeostatic goal attainment. Successful navigation of the stressor tends to resolve the acute stress state.

Yet, the state of stress itself can be conserved and maximized through self-reinforcing negative and positive feedback processes, resulting in a form of stress chronification termed *sustained threat* in the National Institute of Mental Health research domain criteria (RDoC). RDoC operationalizes sustained threat across multiple levels of measurement, from molecules to genes to cells to circuits to behavior. At the behavioral level, sustained threat is manifested by attentional bias to threat, heightened conflict detection, anxious arousal, avoidance, perseveration, helplessness behavior, anhedonia, decreased libido, and punishment sensitivity.^6^ These behavioral indicators are not merely descriptive, but also causally linked, such that each sustained threat behavior may exacerbate other behaviors within the system. As such, one might construe these behavioral indicators as nodes of a sustained threat system whose self-organization may be described as a spiral—that is, a system whose cyclic, reciprocal relations not only preserve but also intensify one another over time (cf., the lower half of Figure 1).^82^ These behavioral indicators are underpinned by stress-related physiological changes, including increased production of neuroendocrine and cytokine molecules that promote arborization of neurons in brain regions mediating sustained threat (e.g., amygdala) and atrophy in self-regulatory brain circuits (e.g., prefrontal cortex, PFC).^7,8^ The spiral of sustained threat occasions a range of physical and psychiatric maladies such as metabolic syndrome, posttraumatic stress disorder (PTSD), chronic inflammation, and depression.^3,9^

**Figure 1.**
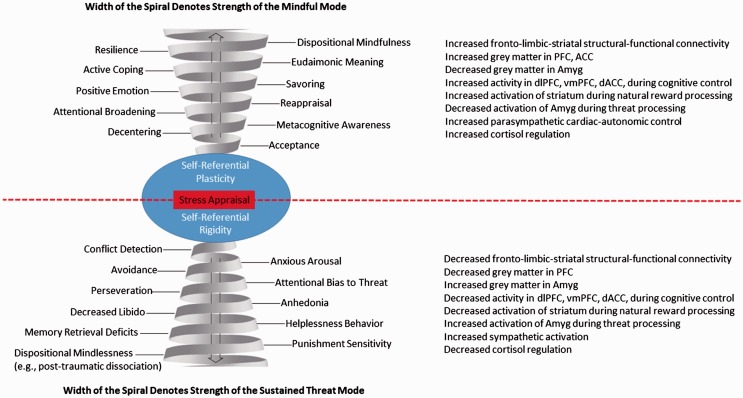
Mindful coping counters the spiral of sustained threat. *Note:* This figure does not posit linear causal linkages between behavioral nodes of each spiral, for indeed, it is likely that there are multiple, recursive relationships between nodes that amplify the strength of the spiral. Also, individual differences in stress responding and propensity toward dispositional mindfulness will create nonequal proportions in the magnitude of mindful vs. stress response, which would be represented by variably sized loops in this spiral model. Unlike the idealized schema depicted, progress toward eudaimonic meaning and resilience is nonlinear, involving multiple iterations of mindful decentering and reappraisal within and across stress coping episodes before a durable sense of well-being is achieved. Although future stressors may result in transient states of acute stress, prolonged mindfulness practice is likely to engender overall upward trajectory of well-being.

If the spiral of sustained threat contributes to pathology via neuroplastic modifications to cognitive-affective brain circuitry, interventions targeting these circuits may reverse this effect, resulting in greater resilience to adversity. In that regard, over the past three decades, mindfulness meditation has gained prominence in medical and behavioral healthcare contexts as a means of stress reduction. Indeed, the vanguard Mindfulness-Based Intervention (MBI), Mindfulness-Based Stress Reduction (MBSR), was^10^ originally developed to treat stress arising from chronic physical and psychological problems. Subsequently, a diversity of targeted MBIs have been developed for select clinical conditions, such as Mindfulness-Based Cognitive Therapy (MBCT),^11^ mindfulness-based relapse prevention,^12^ and Mindfulness-Oriented Recovery Enhancement (MORE).^13^ Evidence suggests that MBIs are promising therapeutic approaches that promote salutary emotional,^14^ cognitive,^15^ and physical health^16^ outcomes. Recent accounts suggest that MBIs produce positive effects via decreased bottom-up stress reactivity^17^ and increased top-down self-regulatory capacity, which emerges from mindfulness-induced enhancements in attention control, emotion regulation, and self-awareness.^18^

Meta-analyses indicate that MBIs significantly reduce stress in clinical^14^ and nonclinical populations.^19^ Yet, while evidence suggests that MBIs may be an effective treatment for stress, this conclusion is based on global assessments of stress as operationalized by self-report measures. In the absence of conceptual and methodological precision, it is difficult to draw mechanistically informed conclusions from such meta-analytic results, though in general MBIs appear to alleviate stress. In contrast to research on general stress, no review has detailed how MBIs modulate specific elements of chronic stress as operationalized by the RDoC construct of sustained threat. The purpose of this article is to provide a selective review of findings from randomized controlled trials (RCTs) and experimental studies suggesting that MBIs can modulate the behavioral and neurobiological mechanisms of chronic stress. Because there is a dearth of research on the ways by which MBIs might modify the sustained threat elements as articulated in the RDoC, this review offers a number of testable hypotheses regarding possible links between mindfulness-related processes and the “RDoC-ian” mechanisms undergirding chronic stress.

## Mindfulness Modulates RDoC Sustained Threat Elements at the Behavioral Unit of Analysis

A growing body of studies demonstrate the effects of MBIs on a wide range of behavioral indicators of the sustained threat (i.e., chronic stress) construct. The following section provides an overview of current findings from studies of multiweek and brief MBIs that specifically address each of the 10 elements of behavioral dysregulation characterizing sustained threat. These elements are discussed in the context of their associated mindful counter-regulatory strategies, which will later be situated and integrated in a novel conceptual framework of mindful self-regulation, the Mindfulness-to-Meaning Theory (MMT).^20^

### Conflict Detection

Conflict detection, the information processing function whereby conflicts in competing goals and action plans are monitored to detect when control must be exerted,^21^ is exacerbated by stress and anxiety^22^ and becomes hyperactive in chronic stress-related psychiatric disorders like anxiety and depression.^23^ Conflict detection is the attentional component most frequently improved by MBIs across studies.^18^ For instance, brief MBIs^24^ and more intensive forms of mindfulness training^25^ improve the efficiency of executive attention during conflict detection. However, some studies of MBIs have failed to observe changes in conflict detection.^26,27^ On the whole, mechanistic evidence and theory suggest that mindfulness-induced improvements in conflict detection are linked with greater emotional acceptance, in that mindful individuals are more likely to accept attentional conflicts and emotions arising from conflicting information.^28,29^ Thus, we hypothesize that by increasing acceptance of the conflicting stimulus, mindfulness may reduce attentional and emotional reactivity to perturbation by the stressor.

### Anxious Arousal

MBIs are effective treatments for anxiety (Hedges’ *g* = .63),^30^ with meta-analytic evidence suggesting MBIs reduce anxious arousal more than active control conditions (Cohen’s *d* = .38).^14^ Hypothetically, the anxiolytic effects of MBIs may stem, in part, from mindfulness practices that train and promote decentering,^31–33^ the process of using attention to “step back” and create psychological distance from negative thoughts and feelings,^34^ facilitating the realization that unpleasant mental events are not necessarily veridical representations of reality.^35^ In support of this hypothesis increases in decentering are associated with decreased anxiety among MBI participants,^32^ and mediate the relationship between effects of MBI on reducing generalized^36^ and somatically focused anxiety.^37^

### Avoidance

Decentering through mindfulness may facilitate metacognitive monitoring of present moment experience without experiential avoidance of aversive cognitions, emotions, or behavioral urges.^38,39^ RCTs have demonstrated that MBIs can reduce emotional^40^ and behavioral avoidance,^41^ and brief mindfulness training has been shown to reduce implicit (but not explicit) measures of experiential avoidance in nonclinical samples.^42^ Studies have also begun characterizing the effects of MBIs on avoidance in persons with PTSD and depression. In clinical trials, MBIs significantly decrease PTSD symptoms^43–45^ coupled with decreases in avoidance.^46,47^ Metacognitive awareness may allow for decreased avoidance of painful emotional content;^48^ indeed, depressed patients randomized to an MBI exhibited increased metacognitive awareness, which allowed them to reflect on rather than avoid thinking about prior suicidal crises.^49^ Similarly, an MBI increased metacognitive awareness of negative thoughts and feelings which facilitated prevention of depressive relapse,^50^ suggesting that metacognitive monitoring may reduce the long-term emotional impact of stressful affective states without avoidance.

### Attentional Bias to Threat

A growing body of evidence suggests that mindfulness training may not only decrease avoidance of negative, stressful stimuli but may also reduce attentional biases to such stimuli—i.e., the maladaptive tendency to pay selective attention to potential threat. In this respect, mindfulness may provide a “middle way” through the extremes of avoidance and hypervigilance for threat by enhancing attentional control, putatively one of the primary mechanisms of mindfulness.^18^ For example, an experimental mindfulness induction reduced negativity bias,^51^ and reduced negativity bias was also observed in previously depressed individuals after completing an MBI.^52^ Similarly, chronic pain patients showed reduced attentional bias to threat following participation in an MBI,^53^ and an RCT of chronic pain patients found that an MBI reduced attentional bias to threat to a significantly greater extent than an active control condition.^13^ Mindfulness may reduce attentional bias to threat by broadening the attentional field to encompass previously unattended contextual features, including neutral and positive stimuli that were previously ignored due to attentional biases.^20^ We hypothesize that attentional broadening may promote well-being in the face of chronic stress by affording an influx of novel data about the stressor context, disrupting cognitive perseveration and providing new input for information processing about the stressor itself.^54^

### Perseveration

Systematic review of the literature indicates that MBIs decrease rumination,^55^ a key form of cognitive perseveration that prolongs and exacerbates sustained threat. Meta-analytic findings demonstrate that mindfulness-induced reductions in perseverative negative thinking mediate the effect of MBIs on alleviating psychological distress.^56^ Mindfulness may decrease perseverative cognition by engaging inhibitory control mechanisms during the process of decentering into the state of metacognitive awareness. In partial support of this contention, mindfulness training has been shown to improve inhibitory control in the face of processing negative emotional information.^57^ Hypothetically, such enhanced inhibitory control over perseverative cognition may then free cognitive resources to fuel more adaptive forms of cognitive reprocessing of the stressor context, such as positively reappraising the stressor as a growth opportunity or source of meaning. Indeed, observational studies have found inverse correlations between reappraisal and perseverative cognitive strategies like rumination,^58^ and in experimental research reappraisal has been shown be associated with decreased cognitive perseveration.^59^ In that regard, a growing body of research indicates that MBIs encourage positive reappraisal.^20^ Cross-sectional studies suggest a positive relationship between mindfulness and positive reappraisal in healthy adults,^60^ patients with psychiatric disorders,^61^ patients with substance use disorders,^60,62^ chronic pain patients,^60^ and meditation practitioners.^63^ Experimental studies and RCTs extend these findings by demonstrating that mindfulness inductions^64,65^ and MBIs^66–68^ can foster positive reappraisal. Although mindfulness may decrease perseverative thought in the absence of reappraisal (such as when mindfulness purportedly leads to “nonappraisal” or a suspension in conceptual activity altogether^69^), reappraisal appears to be a key sequela of mindfulness that generates positive emotions from coping with stress.^70^

### Anhedonia/Decreased Appetitive Behavior

A number of studies demonstrate that MBIs are an effective treatment for depressive disorders (Hedges’ *g* = .59),^30^ reducing depressive symptoms and improving negative affect more than active controls.^14^ Insofar as anhedonia is a key depressive symptom exacerbated by chronic stress,^71,72^ MBIs may be a potent means of countering stress-precipitated anhedonia. In support of this notion, meta-analyses indicate that MBIs promote positive emotions (*r* = .25)^73^ and other studies suggest that MBIs increase reward responsiveness, specifically. For instance, in a RCT, an MBI increased positive emotions and reward experiences in response to pleasant daily life events for individuals with depression histories.^74^ More recently, autoregressive latent trajectory modeling of data from this trial revealed that MBCT drives an upward spiral of positive affective-cognitive states.^75^ Similarly, an RCT demonstrated that an MBI was associated with increases in autonomic^76^ and neurophysiological^77^ responses to natural reward cues among chronic pain patients. Ecological momentary assessments from this trial indicated that relative to an active control condition, MORE was significantly associated with greater likelihood (OR = 2.75) of positive affect regulation (i.e., being able to maintain positive affect or shift affect in a positive direction from moment-to-moment).^78^ One hypothetical explanation for such effects is that mindfulness enhances savoring of natural rewards and the positive emotions that flow from them,^20^ thereby countering anhedonia.

### Decreased Libido

Investigation of the impact of MBIs on sexual desire and arousal has been explored in recent studies. Results from these studies indicate that MBIs increase sexual desire, sexual arousal, and the concordance between subjective and physiological arousal among women with sexual desire and arousal difficulties, including women with histories of childhood sexual abuse.^79–81^ Thus, preliminary evidence suggests that MBIs may improve libido and sexual functioning in women, though no systematic examination of the effects of MBIs on male sexual performance has yet been undertaken. Hypothetically, MBIs may increase libido via mindful savoring, the process of mindfully attending to and appreciating pleasant stimuli as a means of increasing reward responsiveness. Indeed, the practice of savoring has been compared to sensate focus techniques^82^ which aim to amplify pleasure during sex by mindfully sustaining attention on pleasant bodily sensations. While no studies to date have directly examined the relationship between mindfulness training and savoring of sexual pleasure, the aforementioned data on MBIs and sex parallel findings demonstrating that mindful eating can increase food liking,^83,84^ suggesting a promising area of future research. Alternatively, instead of savoring, mindfulness might increase libido by modulating purely bottom-up mechanisms implicated in sexual arousal, such as parasympathetic regulation^85^—an autonomic process known to be tractable to mindfulness training.^86,87^

### Helplessness Behavior

Contrary to stereotypical associations between meditation, stoicism, and passivity, mindfulness has been empirically associated with active coping behavior.^88^ Indeed, mindfulness may be an antidote to learned helplessness.^89^ In support of this hypothesis, RCTs have demonstrated that MBIs increase active cognitive and behavioral coping in cancer patients,^90^ increase problem-focused coping among students,^91^ and increase approach coping while decreasing avoidant coping among stressed individuals.^92^ Additional evidence that mindfulness reduces helplessness behavior comes from an experiment in which a mindfulness induction increased participants’ persistence on a math task after viewing a video clip eliciting a fear response.^93^ Qualitative accounts also support this notion, with recurrently depressed patients reporting that MBI leads to reduced helplessness and a greater sense of control.^94^ Thus, mindfulness may promote active rather than passive responding to stress.

### Memory Retrieval Deficits

Although mindfulness is popularly defined as awareness of present moment experience, a Sanskrit term for mindfulness directly connotes “remembering,”^95^ suggesting that MBIs may remediate chronic stress-induced memory retrieval deficits. A recent meta-analysis supports the semantic connection between mindfulness and memory.^15^ For instance, a brief mindfulness induction positively influenced memory performance,^96^ and participation in a 10-day mindfulness meditation retreat was found to increase working memory.^97^ Mindfulness also appears to influence the type of memory retrieval. Two experimental studies suggest that mindfulness inductions improve recall of positively valenced words.^98,99^ Additionally, participation in MBIs is associated with reduced overgeneral autobiographical memory^100^ (a style of memory that increases depression vulnerability^101^) and increased autobiographical memory specificity.^102,103^ Finally, experimental research suggests that mindfulness training increases false-memory susceptibility, in part due to a decrease in reality-monitoring accuracy, which may result in reduced discrimination between internally generated and externally generated information for memory formation.^104^ The relationship between mindfulness and memory may have important implications for generating eudaimonic well-being. In contrast to hedonic well-being, eudamonic well-being arises from construing a sense of meaning, autonomy, and mastery even under conditions of loss or adversity. Thus, if mindfulness meditation reduces overgeneralized autobiographical memories while enhancing recall of positive information, it may be that MBI involvement encourages greater eudaimonic well-being by facilitating retrieval of memories of successful, active coping with stress and memories of meaningful experiences in spite of stress. This effect may be further potentiated by the effects of mindfulness on reducing discrimination between internally and externally generated memory traces; indeed, if mindfulness makes memory more “malleable,” it may facilitate adaptive reconstrual of past stressors as sources of personal growth and meaning. Through these mechanisms, mindfulness may foster eudaimonic meaning by situating memories of adverse or challenging experiences in a larger autobiographical narrative of growth and resilience—a hypothesis formalized within the MMT and discussed later in this article.^20^

### Punishment Sensitivity

Considering the aforementioned stress-regulatory mechanisms of mindfulness, we hypothesize that MBIs may ultimately undo heightened punishment sensitivity caused by sustained threat. Although to our knowledge no studies have directly examined MBI effects on punishment sensitivity, a large body of experimental and clinical research demonstrates that MBIs reduce the punishing experience of pain.^105,106^ Indeed, laboratory-based studies have shown that MBIs can reduce pain sensitivity^107,108^ and pain intensity^109,110^ during standardized, experimental acute pain inductions, whereas clinical trials indicate that MBIs can reduce chronic pain severity.^67,111^ With regard to nonpainful aversive stimuli, participation in an MBI was associated with reduced respiration rate and enhanced parasympathetic regulation of cardiac defense response to acoustic startle,^112^ and in another study, mindfulness practice was associated with enhanced acoustic startle habituation,^113^ suggestive of decreased punishment sensitivity. On the whole, these studies indicate that in contrast to punishment sensitivity, MBIs promote resilience to aversive, stressful stimuli. In support of this hypothesis, some studies have found MBIs to improve resilience,^100,10^ though effects on resilience in other studies have been equivocal.^114,115^ However, a large RCT of Marines found that a 20-h MBI improved physiological indices of resilience such as heart rate reactivity and recovery after stressful combat-related training operations.^116^ Continued scientific investigation of the impact of MBIs on resilience and punishment sensitivity is needed, as are mechanistic studies to assess the effects of MBIs on neurobiological processes integral to resilient responding to chronic stress.

## Mindfulness Modulates RDoC Sustained Threat Elements at the Circuit, Physiological, and Molecular Units of Analysis

Adaptive outcomes associated with MBIs are evident not only behaviorally but are also apparent within the circuit, physiological, and molecular RDoC units of analysis underpinning chronic stress. Neuroscience studies have accumulated demonstrating effects of MBIs on biological systems relevant to sustained threat, including hypothalamic–pituitary–adrenal (HPA) axis physiology and hormones, and brain circuits involved in attentional function, limbic reactivity, habit behavior, and the default mode network (DMN). Neurobiological correlates of each behavioral MBI outcome are not necessarily isolated within one of these systems, but rather may arise from complex interactions between these systems. The following section reviews experimental studies of multiweek and brief MBIs that address biological domains implicated in sustained threat.

### Attention Network

The attentional network is one of the brain circuits most affected by chronic stress, and likewise by mindfulness practices. Accumulating research shows that MBIs are associated with beneficial neuroplastic changes in the attention network—in particular, regions of PFC and anterior cingulate cortex (ACC) relevant to cognitive control. Meta-analyses indicate that participation in MBIs is associated with greater PFC and ACC activation and thickness.^117–119^ Broad neuroplastic changes in the attention network are linked with a number of specific outcomes relevant to the RDoC construct of sustained threat. For example, MBIs are associated with increased dorsal ACC activity during conflict detection and attentional orienting.^120^ Furthermore, MBIs evoke greater activation in the dorsolateral PFC (dlPFC) that are correlated with reduced attentional conflicts reflected by affective Stroop task performance.^57^ Mindfulness-based enhancements to attentional network structure and function may remediate stress-induced neurocognitive deficits and facilitate regulation of reactivity to stressful stimuli, thereby reducing chronic stress symptoms like anxious arousal, attentional bias to threat, perseverative cognition, memory retrieval deficits, and punishment sensitivity.

### Dysregulation of Amygdala Reactivity

The PFC and ACC engage in top-down regulation of negative emotional reactions to stressful and threatening stimuli instantiated in the amygdala.^121^ Given the amygdala’s central role in fear processing,^122^ anxiety,^123^ and negative emotions,^124^ behavioral improvements in anxious arousal, avoidance, attentional bias, appetitive responding, anhedonia, and punishment sensitivity following from MBIs are likely a function of amygdalar changes. MBIs have been repeatedly associated with salubrious structural and functional changes in the amygdala, including decreased gray matter^125^ and decreased amygdala response to negative emotional stimuli.^126–128^ Further, MBIs alter functional coupling between the amygdala and PFC in salutary ways. For instance, mindful attention to breathing reduces amygdala responses to negative emotional stimuli and enhances PFC-amygdala functional connectivity.^129^ Similar increases in PFC-amygdala functional connectivity were correlated with reduced anxiety in an RCT of an MBI for patients with generalized anxiety disorder.^130^ A recent RCT found that stressed participants randomized into a brief MBI exhibited significant reductions in right amygdala-subgenual ACC resting state functional connectivity (rsFC) that was correlated with decreases in biomarkers of chronic HPA-axis activation, suggesting that MBIs may reduce molecular mediators of chronic stress by downregulating amygdala reactivity.^131^

### HPA-Axis Dysregulation

On a molecular level, anxious arousal associated with chronic stress is driven by hypersecretion of corticotrophin-releasing factor (CRF),^132^ a master endocrine regulator originating in the paraventricular nucleus of the hypothalamus.^133^ Increased CRF, in turn, signals the pituitary gland to over-produce adrenocorticotropic hormone (ACTH), which stimulates the adrenals to release the stress hormone cortisol.^133^ Given that cortisol is the downstream product of stress-induced HPA-axis physiological responses, cortisol has emerged as an indirect marker of improved HPA function associated with MBIs. A recent meta-analysis of five RCTs found statistically significant, small-to-moderate sized effects of MBIs on cortisol secretion, with longer mindfulness-training durations associated with larger effect sizes.^134^ In general, these studies demonstrated decreased cortisol output for participants of MBIs relative to various control condition, although it should be noted that effects of mindfulness on cortisol were inconsistent across studies,^135^ and interpretation of cortisol output measures is quite complex. Effects of MBIs on reducing HPA-axis reactivity are evident in studies that employ experimental stress induction paradigms. For instance, a recent study of adults with generalized anxiety disorder found that MBSR significantly reduced ACTH during the Trier Social Stress Test (TSST) relative to a control condition.^136^ However, some studies have demonstrated increased cortisol reactivity during laboratory stress induction. Participants randomized into a brief MBI showed higher levels of salivary cortisol reactivity to the TSST, though the MBI did reduce self-reported levels of stress.^137^ Finally, in an observational study, state mindfulness during relational conflict was found to predict accelerated cortisol recovery from the conflict^138^ and in a second study, experienced meditators participating in an intensive one-day MBI evidenced reduced expression of histone deacetylase genes and pro-inflammatory genes that were correlated with faster cortisol recovery from the TSST.^139^ Taken together, findings support the hypothesis that mindfulness training may augment adaptive stress responding by first increasing allocation of biological resources for adaptation to the stressful perturbation, and then by reducing stress hormone secretion in the wake of the acute stressor, thereby preventing chronic anxious arousal.

### Habit Systems

Automatized behaviors and habit responses, are thought to be heavily influenced by dopamine signaling in the striatum. Research suggests that chronic stress dysregulates dopaminergic brain regions,^140^ and shifts behavior toward habitual responding.^141^ Indeed, impulsive and compulsive behaviors (like addiction) are correlated with dopaminergic dysfunction in the striatum.^142,143^ By contrast, one seminal study revealed increased striatal dopamine following meditation, suggesting that MBIs may induce neurobiological changes that might support deautomatization of habit behavior.^144^ MBIs are also associated with changes in habit systems during reward processing. Studies have shown that participation in MBIs is associated with reduced functional connectivity between the right caudate and bilateral anterior insula among healthy participants anticipating reward,^145^ and among smokers, decreased striatal responses during habitual responding to cigarette cues coupled with increased striatal responses during processing of natural reward stimuli.^146^ Concerning this latter finding, previous reviews have focused on the extent to which MBIs may influence neural correlates of habitual responding in the context of addiction,^147^ and recent studies suggest that MBIs can, indeed, deautomatize addictive habit responses.^76,148^ Hypothetically, modification of habit systems through MBIs might be associated with improvements in automatized avoidance, perseveration, and helplessness behavior. However, much is left to be discovered about how MBIs may induce change in habit circuits among populations exclusively suffering from chronic stress.

## Transcending Stress Through Self-Referential Plasticity in the DMN

Ultimately, stress is a product of appraising the self-relevance of the potential or actual threat that initiates a primordial defense response in which the self attempts to preserve its own survival.^1^ Theoretically, without a self to defend, stress cannot arise. In that regard, prolonged practice of mindfulness among adepts is believed to result in the experiential realization of the emptiness of the autobiographical self and the interdependent nature of reality.^149^ Such profound (and sometimes terrifying^150^) experiences may lead to a transitory collapse of the perceptual distinction between the subject who appraises and the object that is appraised, a state of nondual awareness^151^ that purportedly nullifies stress reactivity and desire to obtain homeostatic goals.^89^ Thus, if there is no distinction between self and world, and no goal to seek or obtain, stress cannot arise during the transactional process.

A growing body of cross-sectional, observational studies indicates that advanced meditation practices can induce transient experiences of selflessness and concomitant nondual states of awareness that are correlated with alterations in DMN structure and function.^152–154^ The DMN is primarily comprised of the medial PFC, posterior cingulate cortex (PCC), and the inferior parietal lobule—brain regions heavily implicated in resting state activity^155^ and episodic memory processing.^156^ Research has revealed significant reductions in DMN volume,^157^ and aberrant DMN functional connectivity among stressed individuals.^158,159^ Although adept mindfulness practitioners may indeed be able to generate transitory states of consciousness in which the subject–object distinction is diminished in favor of a temporary experience of selflessness, self-related processes like the attribution of autobiographical meaning to experience are crucial for successful adaptation to stress and healthy psychological development in Western society.^160^ Rather than abolishing self-reference entirely, we hypothesize that mindfulness training may induce structural–functional plasticity in the DMN to enhance integration between the DMN and brain networks implicated in cognitive control and salience attribution, thereby facilitating flexible self-referential processing in service of enhanced self-regulation in the face of stress.

In support of this hypothesis, a small but growing body of RCTs suggests that participation in standard eight-week MBI courses is associated with enhanced rsFC between the DMN, the executive control network, and the salience network. An RCT of healthy adults revealed that relative to a control condition, participating in an MBI was associated with increased rsFC between brain regions implicated in salience including posterior insula, regions implicated in executive control including the dorsal ACC, and the right dorsomedial PFC, one of the functional hubs of the DMN.^161^ Similar findings concerning MBI effects on strengthening rsFC between DMN and cognitive control networks have been observed among high stress populations. A recent RCT of stressed, unemployed community adults demonstrated that relative to a relaxation control condition, participation in a brief MBI was associated with increased rsFC between the default mode hub of the PCC and the dlPFC—a brain region that subserves cognitive control.^162^ A small RCT of combat veterans with PTSD found that participation in an MBI was associated with significant increases in rsFC between PCC, dlPFC, and dorsal ACC following therapy which were significantly correlated with improvement in avoidant and hyperarousal PTSD symptoms.^47^

In general, enhanced rsFC between default mode, executive-attentional, and salience networks may allow for improved self-regulation of stress-related emotional perturbations and maladaptive habits.^147^ Yet, given that the DMN is implicated in self-referential and memory retrieval processes,^163^ MBI-induced structural and functional changes in this network may ameliorate memory deficits and improve autobiographical specificity. Ultimately, MBIs may promote salutary neuroplastic changes in the DMN integral to resilience, updating default, threat-laden schemas of self and world into more adaptive modes of self-reference. This final exploratory hypothesis is considered later.

## Reversing the Spiral of Sustained Threat Through the Shift from Mindfulness-to-Meaning

Functional connectivity data suggest that mindfulness and related meditation practices may induce a form of self-referential plasticity, hypothetically facilitating flexible reconfiguration of the self-schema in its adaptation to the world through the exercise of cognitive control mechanisms. This process may in turn result in a transformation of the contextual meaning of experience, including experiences of adversity. Teasdale and Chaskalson^164^ state:

mindfulness is characterized by configurations of cognitive processing in which working memory for implicit, intuitive meaning plays a central role; when mindfulness transforms suffering by changing the way experience is processed or viewed, the integration of information into new patterns within this working memory plays a central role (p. 109).

This notion is fleshed out in the temporally dynamic, causal model specified by MMT, which posits that the practice of mindfulness augments flexible cognitive control and thereby facilitates appraisal and meaning-making processes as the culturally embedded, autobiographical self navigates through life’s challenges.^20,54^

The MMT model (see Figure 1) initiates with mindful acceptance of the stressor by attending to its presence without registering the stressor or one’s initial reaction to it as being in conflict with a more flexible frame of self-reference. Acceptance allows one to recognize reactivity without conflict, suppression, or self-blame, and cues the need to decenter from maladaptive cognitive-emotional reactions. Through decentering, the mindfulness practitioner engages in a “letting go” of the stress appraisal and its attendant anxious arousal by viewing anxiogenic thoughts and feelings from a metacognitive perspective, in which one monitors and deidentifies from mental events, without experiential avoidance. Over time, decentering from stress appraisals into the state of metacognitive awareness may disrupt default activation of threat-related schemas and scripts, gradually extinguishing habitual conditioned stress responses via the repeated process of focusing attention on one’s experiential relation to the conditioned stimulus rather than fulfilling the conditioned response. In so doing, mindfulness may dissolve rigid patterns of defensive self-reference, and broaden the scope of information processing from a narrowed attentional bias toward threat to encompass an expanded set of positive and negative life events. Thus, instead of perseverating on exclusively negative content, situational appraisals are reconfigured in working memory via inclusion of a more balanced set of positive and negative events that have entered consciousness through mindfulness. The reconfiguration process preserves primary appraisals while reconfiguring secondary appraisals—the immediate negative impact of an event is acknowledged, but is then integrated into a positive reappraisal in which adverse life events are seen to lead to personal growth or meaning.

Such reappraisals then generate positive emotions to fill the void of anhedonia, which in turn, become a target for savoring through mindfully attending to and appreciating pleasant experience—countering decreased libidinal drive through an enhanced ability to obtain a sense of reward and hedonic well-being from salutary objects, events, and experiences in the natural environment. Motivated by positive emotions, the mindfulness practitioner may overcome tendencies toward helplessness behavior and instead engage in active coping with the stressor, resulting in experiences of efficacy and success. As time unfolds, rather selectively retrieving overgeneral memories of threat, helplessness, and failure, mindfulness may prime memory for instances of successful stress coping, which can then be integrated into a eudaimonic autobiographical narrative marked by the theme of meaningfulness in the face of adversity. In so doing, recurrent experiences of navigating stress are likely to accrue into the stable belief that the practitioner has the capacity to live in personally meaningful ways and experience an enduring well-being regardless of challenging circumstances. As such, reframing stressful events as inherently meaningful for personal growth and development is a key means of developing resilience out of the encounter with adversity, insofar as this cognitive process reduces sensitivity to the punishing aspects of stressors and promotes stress recovery. As the linkages between nodes of the upward spiral from mindfulness to meaning grow stronger, the consolidation of this new semantic network into long-term memory induces cognitive plasticity resulting in a greater dispositional propensity toward mindful responding in the future—in direct opposition to the “mindless,” automatized, and dissociated mode characteristic of sustained threat.

## Conclusion

For nearly three decades, MBIs have been heralded as promising means of alleviating chronic stress. Now, a substantial body of scientific evidence has amassed to indicate that the practice of mindfulness can modulate a range of behavioral and neurobiological elements implicated in adaptive stress coping. Such mechanistic findings can inform the treatment development process to optimize the next generation of MBIs for greater therapeutic efficacy. However, given the great diversity of MBIs (which include a multiplicity of therapeutic techniques), more research is needed to determine whether mindfulness is the active ingredient underlying the aforementioned treatment outcomes and mechanisms. Also, systematic investigation is required to identify boundary conditions that delimit the effects of MBIs on the elements of sustained threat, for certain biobehavioral factors may indeed be intractable to mindfulness training. In time, science may elucidate the biobehavioral pathways by which mental training interventions like mindfulness restructure dynamical cognitive-affective-biological systems implicated in health and disease via transforming appraisals of self and world. For though stress is ultimately a transactional, cybernetic process, mindful coping may provide the opportunity to find meaning in adapting to a challenging and changing environment.
